# Short-Term Outcome of Inpatient Treatment for Adolescents with Anorexia Nervosa Using DSM-5 Remission Criteria

**DOI:** 10.3390/jcm10143190

**Published:** 2021-07-20

**Authors:** Dunja Mairhofer, Michael Zeiler, Julia Philipp, Stefanie Truttmann, Tanja Wittek, Katrin Skala, Michaela Mitterer, Gabriele Schöfbeck, Clarissa Laczkovics, Julia Schwarzenberg, Gudrun Wagner, Andreas Karwautz

**Affiliations:** Eating Disorders Unit, Department of Child and Adolescent Psychiatry, Medical University of Vienna, 1090 Vienna, Austria; dunja.mairhofer@meduniwien.ac.at (D.M.); michael.zeiler@meduniwien.ac.at (M.Z.); julia.philipp@meduniwien.ac.at (J.P.); stefanie.truttmann@meduniwien.ac.at (S.T.); tanja.wittek@meduniwien.ac.at (T.W.); katrin.skala@meduniwien.ac.at (K.S.); michaela.mitterer@meduniwien.ac.at (M.M.); gabriele.schoefbeck@meduniwien.ac.at (G.S.); clarissa.laczkovics@meduniwien.ac.at (C.L.); julia.schwarzenberg@meduniwien.ac.at (J.S.); gudrun.wagner@meduniwien.ac.at (G.W.)

**Keywords:** anorexia nervosa, adolescents, inpatient treatment, treatment evaluation, remission criteria, DSM-5

## Abstract

This study evaluated the short-term outcome of a multimodal inpatient treatment concept for adolescents with anorexia nervosa (AN). In this prospective observational study, a cohort of 126 female adolescents with AN (age range: 11–17, mean age: 14.83) was longitudinally followed from admission to discharge (average duration of stay: 77 days). We used gold-standard clinical interviews and self-report data, as well as DSM-5 remission criteria, to evaluate the treatment outcome. From admission to discharge, body-mass-index (BMI) significantly improved by 2.6 kg/m^2^. Data from clinical interviews and self-reports yielded similar improvements in restraint eating and eating concerns (large effects). Lower effects were observed for variables assessing weight/shape concerns and drive for thinness. At discharge, 23.2% of patients showed full remission of AN, 31.3% partial remission, and 45.5% no remission according to DSM-5 criteria. Differences in remission groups were found regarding AN severity, age at admission, and use of antidepressant medication. Living with both parents, longer duration of inpatient treatment and the use of antipsychotic medication were significantly associated with higher BMI change. The findings provide evidence for the short-term effectiveness of our inpatient treatment concept. We recommend using DSM-5 based remission criteria to evaluate the treatment outcome to improve the comparability of studies.

## 1. Introduction

Anorexia nervosa (AN) is a serious and complex psychiatric disorder characterized by self-directed severe weight loss, intense fear of weight gain, disturbed body image, and secondary medical problems associated with malnutrition involving a vast variety of bio-psychosocial influencing risk and maintenance factors [1,2,3,4,5]. AN has the highest mortality rate [6,7] and health system costs [8,9] among all psychiatric disorders with a reported chance of full recovery still less than 50% [10,11,12,13,14]. Especially female adolescents between 15 and 19 years are affected by AN with a postulated decrease in the age of onset [7,15,16,17,18]. Recent reviews reported a better prognosis in children and adolescents compared to patients with first-onset AN in adulthood, which emphasizes the need for early recognition, diagnosis and treatment to prevent relapse and a chronic, protracted, or enduring course of this disorder [19,20,21,22,23,24]. The complex phenomenology of this illness and its long enduring course of over 20 years in more than half of those affected, requires specially adapted treatment concepts to increase remission and recovery rates [1,3,5,24]. Current treatment guidelines recommend a multimodal stepped care model for adolescents suffering from AN [25,26]. The intensity of the treatment setting should be adapted to the severity of the disorder, taking into account comorbidities and medical complications. Outpatient psychological/psychotherapeutic treatment involving family members (e.g., family-based treatment, parent-focused treatment, systemic family therapy, enhanced cognitive-behavioral therapy, focal psychodynamic therapy) should be the first step, eventually followed by day clinic and inpatient treatment [1,3,19,25,26]. The current recommendations for consideration of inpatient treatment include a body-mass-index (BMI) below the 3rd percentile, rapid weight loss, low energy intake, refusal to drink, medical complications, severe psychiatric comorbidity, dysfunctional family interactions, and insufficient response to outpatient treatment [25,26,27]. The main goals of inpatient treatment are medical stabilization and nutritional rehabilitation with an additional focus on improvement in eating disorder symptoms and general psychopathology initiating a longer-term psychotherapeutic process [3,13,25,26].

The value of inpatient treatment and the optimal length of an inpatient stay are subjects of controversial debate, because of high drop-out, relapse and readmission rates as well as high health system costs [14,28,29]. This is why several studies evaluated the short- and long-term effectiveness of inpatient treatment compared with day-clinic and outpatient treatment settings [14,29,30,31,32]. Additionally, research on predictive factors that influence the length of inpatient stay getting into focus in more recent years [33,34,35], partly because of the great variation across countries, with a postulated longer duration in Europe (105.6 days) compared to the United States (49.1 days) [36].

However, several previous studies and reviews confirm the effectiveness of inpatient treatment for adolescent with AN regarding an increase in body weight and decrease in eating disorder symptoms from admission to discharge [37,38,39,40,41,42]. In contrast, the effects on core cognitive-psychological symptomatology of AN such as body dissatisfaction, drive for thinness, and weight and shape concerns were much more diverse, with some studies having found no significant improvements and some with low effect sizes of inpatient treatment on these outcomes [31,43,44,45]. It is worth noting that many of these studies mainly used self-report assessments such as the Eating Disorder Examination—Questionnaire (EDE-Q) or the Eating Disorder Inventory-2 (EDI-2). To date, only few studies used investigator-based interviews such as the Eating Disorder Examination Interview (EDE) to evaluate the inpatient treatment outcome [29,46,47,48], although this is considered as the gold-standard in eating disorder assessment [49,50,51,52]. However, inconsistencies regarding treatment effects raised by previous studies might be due to differences in the type of report (clinical rating vs. self-report). Passi and colleagues [50] compared outcomes obtained with the EDE and the EDE-Q in adolescents with AN and found higher overall scores in the EDE-Q than in the EDE. A possible explanation for these discrepancies may be that patients tend to be more honest about the severity of their symptoms in a self-report format and tend to underreport their symptoms in a clinical interview [49]. Therefore, it is important to include both the clinician and the patient perspective when evaluating the outcome of an inpatient treatment setting to minimize the risk for potential misinterpretations by using either self-reports or expert ratings only.

Another point that limits the comparability of previous studies is the heterogeneity of used outcome parameter and varying definitions for “favorable” vs. “unfavorable” treatment outcomes as highlighted by recently published reviews and studies [21,38,53,54,55,56]. Concerning the lack of internationally clearly defined outcome criteria for AN, the fifth edition of the Diagnostic and Statistical Manual of Mental Disorders (DSM-5) [57] formulated specific criteria for full, partial and no remission of AN. To date, these remission criteria were hardly used to evaluate the outcome of inpatient AN treatment. Thus, consistently using these criteria in future studies would improve the comparability of outcomes across different treatment approaches.

As many patients with AN show an unfavorable treatment outcome, several previous studies focused on identifying underlying positive or negative prognostic factors to increase insight into the mechanisms of AN in order to improve current treatment interventions [37,40,58,59,60,61,62,63]. However, the current evidence does not yet provide a sufficient picture on the factors relevant for favorable treatment outcome on the one hand [22,24,54,64,65], and optimal length of inpatient stay on the other hand [33,34,35,36], primarily due to the inconsistent results and high heterogeneity of the methods used.

Regarding the treatment outcome, lower BMI at admission and discharge predicted worse outcome in most studies [37,61,64,66,67], while BMI at discharge showed less impact on treatment success in adolescents when a structured outpatient treatment was implemented directly after discharge [13,14]. Additionally, variables such as age at admission, age of eating disorder onset, the duration of illness, eating disorder severity, familial problems, weight suppression, previous inpatient treatment, and early treatment response were identified as possible positive or negative prognostic factors on treatment outcome in previous studies and reviews [22,61,64,65,68]. Furthermore, the impact of restrictive vs. binge-purging subtype of AN as well as comorbid psychopathology such as depression or anxiety were considered. While some studies showed that a binge-purging subtype [64,65,67,69] as well as the presence of comorbid psychiatric disorders [40,64,70,71] were associated with worse treatment outcome, other studies failed to verify these findings [72,73,74,75]. Sociodemographic, clinical, and therapeutic variables could be found that predicted the length of hospital stay including age and BMI at admission [33,34,35,36,46]. Lascar et al. [33] found that a longer duration of inpatient stay was predicted by older age and lower BMI at admission, longer duration of illness, high severity of eating disorder symptoms, binge-purging subtype, readmissions, enteral or a combination on parenteral and oral nutrition during hospitalization, involuntary treatment, and hospitalization away from home. A shorter hospital stay was predicted by a greater self- confidence, oral re-nutrition, oral nutritional supplementation on admission, hospitalization in an urban area. In addition, there is controversy about whether psychopharmacological treatment has an impact on treatment outcome in adolescent patients with AN [76,77,78]. Indeed, the evidence on the effects of psychopharmacotherapy, such as antidepressants, second-generation antipsychotics or anxiolytics, on the treatment outcome in adolescents or adults with AN is inconsistent, with some studies reporting a positive effect on weight gain and psychological symptoms [79,80,81,82,83], while others found no benefit compared to the placebo [70,81,84,85,86,87].

Despite the growing literature on potential predictors for favorable vs. unfavorable treatment outcomes in adolescent AN, there is still an urgent need for further studies that can shed light on the effectiveness of inpatient treatment approaches in terms of specific factors that may contribute to better treatment responses and remission.

Thus, the aim of the present observational study was to examine and evaluate the short-term effectiveness of a multimodal specialized inpatient treatment concept for adolescents affected by AN as a measure of quality assurance. We used both, clinical interviews and self-reports and additionally evaluated the treatment outcome according to the DSM-5 defined criteria for remission. Furthermore, we explored whether different sociodemographic and clinical characteristics as well as psychopharmacological treatment can predict full or partial remission of AN symptoms, BMI change and duration of inpatient stay. The finding from this study will expand the previous literature on response to inpatient treatment and predictors for the short-term treatment outcomes in adolescent inpatients with AN.

## 2. Materials and Methods

### 2.1. Study Population and Procedure

The participants of this study were recruited within a prospective, observational longitudinal investigation that aimed at evaluating the inpatient treatment concept for adolescents with AN (described in detail below 2.2) at the Eating Disorders Unit of the Department of Child and Adolescent Psychiatry at the Medical University Hospital of Vienna. This study was conducted between 2007 and 2019. All adolescent patients aged between 11 and 17 years, with a definite diagnosis of AN according to the 10th revision of the International Statistical Classification of Diseases and Related Health Problems [88] with a BMI below 17.5 kg/m^2^ (AN restrictive type F50.00, AN binge-purging type F50.01), who were admitted to the Eating Disorders Unit during this period were eligible for inclusion.

Patients as well as their parents gave written informed consent. Ethical approval for this study was granted by the Ethics Committee of the Medical University of Vienna (EK493/07; 09.10.2007).

**Figure 1:**
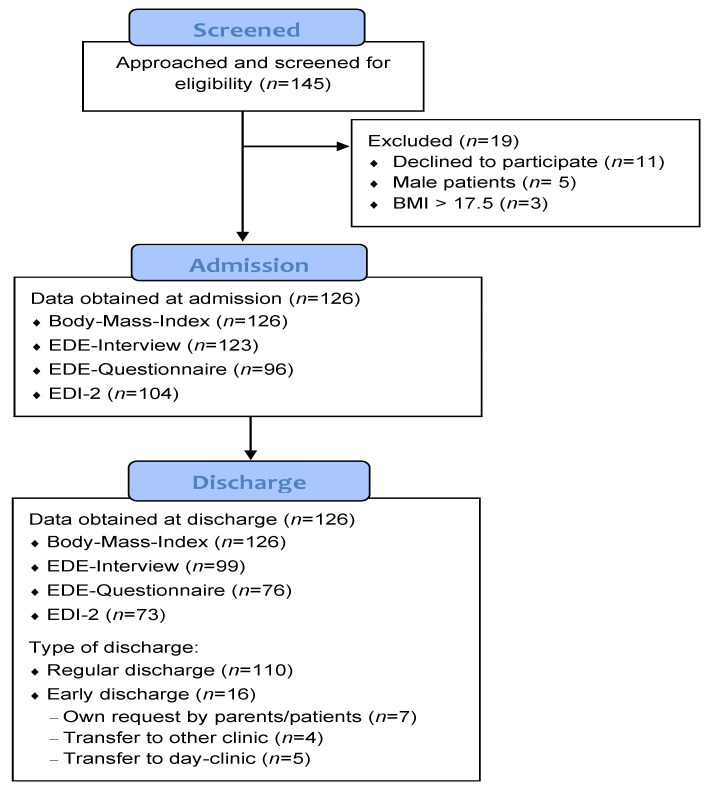
Participants’ flow.

In total, 145 adolescents were approached, of whom 11 patients declined to participate. Male patients (*n* = 5) and patients suffering from atypical AN (*n* = 3) were excluded. Thus, the final sample comprises 126 female adolescents with AN who underwent the Viennese Treatment—Concept for Adolescents Suffering Anorexia Nervosa (‘*ViTAA*’) during their inpatient treatment. The patients completed a standardized assessment of clinical parameters, set of questionnaires, and clinical interviews (see below) at admission (T0) and at discharge (T1). Figure 1 presents the participants’ flow.

### 2.2. Treatment Concept (‘ViTAA’)

The Eating Disorders Unit at the Department of Child and Adolescent Psychiatry at the Medical University Hospital of Vienna provides highly specialized inpatient and outpatient services for children and adolescents affected by eating disorders—especially AN. Our department receives referrals from general practitioners, outpatient clinics, and other hospitals (pediatric, psychosomatic wards) in the Vienna and surrounding area, but also admits patients from other regions of Austria upon request. The department has had in-depth experience in treating patients suffering from eating disorders for many decades and has incorporated evidence-based treatment concepts and recommendations. AN treatment according to the *National Institute for Health and Care Excellence guidelines* (NICE) [25] and *Joint German Guidelines* (S3-Leitlinie) [26] is based on a multimodal stepped care model including outpatient, day clinic and inpatient service which are continuously evaluated and supplemented by new approaches. Inpatient treatment is only considered, if criteria like BMI <3rd percentile, rapid weight loss, low energy intake, refusal to drink, medical complications, severe psychiatric comorbidity, dysfunctional family interactions like high parental criticism or insufficient response to outpatient treatment are met [25,26,27].

The multimodal disorder-specific and age adapted concept (‘*ViTAA*’) is operated by a specially educated multi-disciplinary team that consists of child and adolescent psychiatrists, child and adolescent or pediatric nurses, clinical psychologists, clinical nutritionists, physiotherapists, occupational therapists, psychotherapists, social workers and social pedagogues. The treatment concept includes 35 h per week of different psychotherapeutic and skill-based interventions such as individual psychotherapy, cognitive group therapy, psychodynamic therapy, art and music therapy, skills training, cooking training, and nutritional counseling groups. The treatment concept is explained by the case manager to both parents and patients, to which they consent at the beginning of inpatient treatment. Depending on the somatic condition and severity of the illness (e.g., BMI percentile at admission, medical complications such as risk of developing a refeeding syndrome, electrolyte abnormalities, severe sinus bradycardia, pericardial effusion, orthostatic hypotension) the treatment concept aims at an increase of body weight between 300–1000 g per week. Weight checks are carried out according to the treatment phase from daily to once a week. The multimodal inpatient treatment program (‘*ViTAA*’) encompasses five state-adapted phases of AN treatment with each phase having its own specific goals (e.g., diagnostic, weight gain, stabilization, re-integration); the patients’ self-responsibility is successively increased over the treatment phases. A more detailed description of the treatment phases is provided in the Supplementary Table S1.

### 2.3. Measurements and Instruments

In order to evaluate the effectiveness of this inpatient treatment concept, assessments of eating disorder pathology using gold-standard clinical interviews and self-report questionnaires were carried out by a specially trained research team including medical doctors, clinical psychologist and medical and psychology students who were supervised by professionals in the field and were not a part of the active treatment team. Outcome variables were collected at admission and shortly before discharge. The following clinical patients’ characteristics were obtained in the clinical interview, questionnaires and clinical records: BMI and age- and sex- adjusted BMI percentiles at admission and discharge, premorbid weight (kg) and height (m), weight suppression (defined as the difference between maximum and minimum weight), illness duration, age of AN onset and AN subtypes (restrictive vs. binge-purging), AN severity (according to DSM-5 BMI percentile cut-offs), medication use and the type of referral to our department, as well as the length of inpatient stay. Furthermore, psychiatric comorbidity of the patients was assessed and diagnosed by child and adolescent psychiatrist according to ICD-10 [88]. Sociodemographic data obtained in this study included the patients’ living situation, number of siblings, type of school attended, parental age, parental education background, and parental marital status.

#### 2.3.1. EDE & EDE-Q

The Eating Disorder Examination Interview (EDE) [89] is a semi-structured, investigator-based interview that assesses eating disorder psychopathology. This interview is regarded as the gold-standard of eating disorder assessment worldwide. The Eating Disorder Examination- Questionnaire (EDE-Q) [90] is the corresponding self-report instrument. Both instruments include the same 22 items assessing core symptoms of eating disorders, which are rated on a 7-point Likert-scale from 0–6. Item ratings are summed up to four subscales (Restraint Scale, Eating Concern Scale, Weight Concern Scale, Shape Concern Scale) and a global score. We included both the EDE and EDE-Q, as previous studies have shown that clinician rating and self-reports sometimes diverge although correlations between these measures may be high [49,50] probably because adolescents with eating disorders tend to deny or understate their symptoms. Thus, including both perspectives will help to evaluate whether the treatment is equally effective from the clinician’s and patient’s point of view.

#### 2.3.2. EDI-2

The Eating Disorder Inventory-2 (EDI-2) [91] is a widely used multidimensional self-report questionnaire that assesses different cognitive and behavioral characteristics in patients with different types of eating disorders. We additionally included the EDI-2 as it also assesses aspects of the personality and temperament known to be pronounced in eating disorder patients (e.g., perfectionism, impulse regulation), which is not covered in the EDE/EDE-Q. Moreover, in previous studies, treatment effects evaluated with the EDI-2 were lower as when evaluated with the EDE [92]. It comprises 91-items which are rated on a 6-point Likert-scale ranging from 1–6. Item ratings are aggregated to 11 subscales as well as a total score representing the overall eating disorder psychopathology.

#### 2.3.3. Definition of Remission

According to the DSM-5 [57] criteria for remission, patients were categorized in the full remission group if no diagnostic criterion for AN was met at discharge. Patients were categorized into the “Partial remission” group being weight-restored (BMI ≥ 10th sex- and age-specific percentile, c.f. criterion A) but either criterion B (intense fear of gaining weight, becoming fat or behavior to that interferes with weight gain) or criterion C (disturbances in self-perception of weight and shape) was still met. Patients were categorized into the category “No remission” if the BMI was still below the 10th percentile. Relevant information to build these remission groups were taken from weight/height measures (criterion A) and the EDE clinical interview. Criterion B was met if at least one of the following symptoms assessed with the EDE were present: fear of weight gain (rating ≥ 4), restriction in dietary intake (<1.200 kcal/day), self-induced vomiting, use of laxatives or diuretics on ≥15 days during the preceding month or excessive exercise on ≥15 days during the preceding month. Criterion C was evaluated based on the following items from the EDE: Feeling of being fat, importance of weight or importance of shape (rated with ≥4 during the preceding three months).

### 2.4. Statistical Analyses

The statistical analyses were performed using SPSS 26.0 statistic software (IBM Corp. Released 2019, Version 26.0. Armonk, NY, USA). The global significance level for statistical tests was set to 0.05 and all significance tests were 2-tailed. Bonferroni-corrected significance levels were used when subscales of instruments were analyzed to account for multiple comparisons. All outcome data were visually inspected and checked for normal distributions.

First, we used paired-sample *t*-tests to analyze changes in the BMI, BMI percentile, EDE, EDE-Q and EDI-2 global and sub scores between admission and discharge. We calculated pre-post effect sizes in terms of Cohens *d* (*d* ≥ 0.8 = large effect; *d* ≥ 0.5 = medium effect, *d* < 0.2 = small effect) [93] including 95% confidence intervals. Furthermore, we used general linear models (for continuous variables) and Chi^2^-tests for categorical variables to determine whether remission groups (full, partial, no remission) differed with regard to sociodemographic, clinical and therapeutic characteristics including age at admission, BMI (percentile) at admission, age of eating disorder onset, duration of illness, weight suppression, EDE, EDE-Q, EDI-2 global scores at admission, any psychiatric comorbidity, medication use, duration of inpatient stay, and family status. Tukey post-hoc tests were calculated to explore pairwise comparisons between the remission groups.

We further used univariate and multiple linear regression analyses to evaluate whether potential sociodemographic, eating disorder specific clinical variables and psychopharmacotherapy can predict the change in BMI and the duration of inpatient treatment. We selected predictors that have been found relevant for the treatment outcome in previous studies and/or their impact have been controversially discussed (either positively or negatively influencing the outcome) e.g., [24,54,64] see introduction session for more details. All predictors with *p* < 0.10 in the univariate linear regression models were considered in the multiple regression analyses. This approach was chosen in accordance to Hosmer and Lemeshow [94] who recommended to also consider variables that just failed to reach statistical significance in the univariate models as candidate for the multiple regression model to avoid loss of information. In the multivariate regression analyses, all predictors were entered simultaneously to the model.

#### Power Analysis

We performed a post-hoc power analysis to determine the minimum effect size necessary to detect a statistically significant improvement in eating disorder symptoms with the reached sample size (*n* = 126 for BMI and *n* = 96 for EDE, *n* = 67 for EDE-Q and EDI-2 analyses). Using a *t*-test for paired sampled and a significance level of 0.05, small to medium effects (Δ 0.29–0.40) can be detected with a power of 90%.

## 3. Results

### 3.1. Sociodemographic and Clinical Characteristics of the Study Sample

During the study period, 126 female adolescents from those consecutively admitted participated in this study. The availability of outcome data obtained from the clinical interview or self-report questionnaires is shown in Figure 1. A detailed description of the study population is provided in Table 1. The mean age of the patients at admission was 14.83 (SD: 1.56) with an average AN duration of 13.48 months (SD: 10.42). The majority of patients (88.1%) were diagnosed with AN restrictive type. The mean BMI-percentile at admission was 1.47 (SD = 3.41) and BMI was 14.40 (SD = 1.32) on average. Any psychiatric comorbidity was diagnosed in 64.3% of patients. Any antipsychotic medication (olanzapine, aripiprazole) was prescribed in 62.7%, any antidepressant medication (mirtazapine, sertraline, fluoxetine) in 43.7%, anxiolytics (alprazolam, lorazepam) in 19.8% and nutritional supplements (thiamine, colecalciferole, cobalamine, pyridoxine, vitamins, zinc) in 100% of cases. Most adolescents (65.1%) lived with both parents in the same household and most parents were married or lived in a partnership (mothers: 74.2%, fathers: 78.5%). A total of 43.6% of fathers and 37.0% of mothers had a university degree.

### 3.2. Change in BMI and Eating Disorder Psychopathology

The changes in BMI, EDE, EDE-Q, and EDI-2 scores from admission to discharge are shown in Table 2. The BMI and BMI percentile increased significantly by Δ2.6 (BMI) and Δ11.3 (BMI percentile) on average with discharge weights between 32 to 52 kg and large effect sizes (*d* = 2.01 and *d* = 1.07). Regarding the EDE and EDE-Q statistically significant improvements were observed for the total scores and all sub scales (all *p*-values < 0.001). The effect sizes were largest for the sub scale “Restraint” (EDE: *d* = 1.06; EDE-Q: *d* = 0.94) and lowest for the sub scale “Weight concern” (EDE: *d* = 0.49, EDE-Q: *d* = 0.49) and “Shape concern” (EDE: *d* = 0.35; EDE-Q: *d* = 0.42). Regarding the EDI-2 statistically significant improvement were observed for the total score and the sub scales “Drive for Thinness”, “Interpersonal Distrust”, “Interoceptive Awareness”, “Impulse regulation”, and “Social Insecurity” with all effect sizes for these scales being in the small to medium range (*d* between 0.33 and 0.45). Of note, EDE, EDE-Q and EDI-2 total scores at admission and discharge were highly correlated (between *r* = 0.62 and *r* = 0.79); correlations of EDE/EDE-Q scores with EDI-2 subscores were in the medium range only. The bivariate correlations are provided in Table S2 and Figure S1 in the Supplementary Material.

### 3.3. Outcomes Related to Remission According to DSM-5 Criteria

At discharge, 23.2% (*n* = 23) of the patients showed “Full remission” of AN-symptoms, 31.3% (*n* = 31) showed “Partial remission”, and 45.5% (*n* = 45) showed “No remission” according to the definition published in the DSM-5 manual. Differences between remission groups regarding sociodemographic and eating disorder specific clinical characteristics of the patients are given in Table 3. Patients with “No remission” at discharge were significantly older, had lower BMI percentiles and higher levels of eating disorder pathology at admission and later eating disorder onset compared to patients with “Full and/or Partial remission”. Moreover, more patients in the “Partial remission” group used antidepressant medication compared to patients in the “Full remission” group.

### 3.4. Predictors for Change in BMI and Length of Inpatient Stay

We entered postulated predictors discussed in the current literature into univariate linear regression models to predict the change in BMI and the duration of inpatient stay (Tables S3 and S4 in the Electronic Supplementary Material). The results of the multivariate regression analyses are shown in Table 4. Predicting the pre-post change in BMI lower BMI at admission, longer duration of inpatient stay, living with both biological parents and the use of antipsychotic medication were significantly positively associated with greater BMI increase with the model explaining 58% of the variance. Furthermore, a lower BMI at admission, a higher EDE total score at admission and the use of anxiolytic medication significantly predicted the duration of inpatient stay (model explaining 26% of the variance).

## 4. Discussion

The main aims of this study were to evaluate the effectiveness of a specialized multimodal inpatient treatment concept for adolescents with AN considering clinician’s ratings and self-reports as well as the DSM-5 remission criteria to assure the quality of the treatment. We further identified sociodemographic, clinical and psychopharmacological predictors of the treatment outcome and the length of inpatient stay. We observed a significant increase in BMI and BMI-percentiles and a significant decrease in eating disorder symptoms between admission and discharge, indicating the effectiveness of our multimodal inpatient treatment program on core-symptoms.

The highest effect sizes were found for increase in BMI which are comparable to the effects found for other inpatient treatments [14,31,37,39,40,41,43,46,75]. Similarly, the effects on restraint eating and eating concerns are in line with findings from previous studies [14,31,37,40,43,44,45]. Noteworthy, lower effects (some of them having reached statistical significance, some of them not) were observed for outcomes assessing weight/shape concerns, drive for thinness, body dissatisfaction, ineffectiveness, asceticism, maturity fears as well as for outcomes related to temperament (e.g., perfectionism, impulse regulation). There are two possible explanations for this finding. First, underlying psychological traits and core beliefs may need more time for change and thus, a change may be difficult to observe during a relative short period of inpatient treatment. Second, our inpatient treatment concept focuses particularly on somatic stabilization, nutritional rehabilitation and changes in eating behaviors, while some therapeutic processes targeting body image and temperament factors just start during the inpatient phase and will be intensified during the subsequent outpatient psychotherapy.

To date, assessments of eating disorder severity involving both clinical experts’ and patients’ perspectives have rarely been applied to evaluate inpatient treatments for adolescents with AN in previous studies [50,52]. A parallel use of EDE and EDE-Q measures point to similar effects for clinical expert-ratings and self-reports. To note, a previous study has reported higher overall EDE-Q scores compared to the EDE [50], while EDE-Q scores tend to be slightly lower than EDE scores in our sample, which may indicate a potential underestimation or denial of symptoms when assessed by self-report. Nevertheless, the similarity in scores and effects demonstrate that self-report instruments for assessing AN psychopathology in adolescents have a relatively good informative value.

Up to now, only a few studies have used and examined the inpatient treatment outcome considering the DSM-5 criteria for remission. In our study, more than half of the patients showed at least partial remission of their AN-symptoms at discharge what can be regarded as success considering the short treatment time. However, less than half of the patients did not show remission. With regard to the unmet weight criterion, it must be mentioned that discharge sometimes takes place earlier if basic nutritional stabilization is achieved and an intensive and structured treatment in an outpatient setting is available subsequently and can be accessed immediately after discharge.

Due to the lack of studies having used DSM-5 criteria for remission, the comparability of remission rates with other studies is limited. To our knowledge, only the study by Graell et al. [95] is comparable to a limited extent, as their analyses combined outcomes from mixed settings. After 6 months, 7% of the study population showed partial remission and no participant full remission of AN-symptoms. Other studies used different approaches to assess individual treatment success, such as remission criteria defined by the Morgan Russel scale [96] or the concept of clinical significant outcomes according to Jacobs and Truax [97]. For instance, Herpertz-Dahlmann et al. [14] reported that 25% of patients achieved a good, 19% an intermediate and 56% a poor outcome following inpatient treatment according to the Morgan Russell scale. Schlegl et al. [40], for example, found that almost 70% achieved a clinically significant or a reliable change in the EDI-2 self-report. Future studies may profit from consistently reporting remission rates defined by DSM-5 to improve comparability between effects from different treatment approaches.

Moreover, significant differences between the three remission groups were found regarding various baseline variables. Fully or partially remitted patients were at younger age with an earlier onset of the eating disorder, had a higher BMI percentile at admission, and less eating disorder symptoms. This goes in line with the current literature that younger patients with a higher BMI at admission, shorter illness duration, and lower eating disorder severity have a better treatment response, outcome, and prognosis [37,59,64,65,66,68]. Thus, our results confirm the current recommendations of early recognition, diagnosis and treatment start to prevent an enduring course also in patients without psychiatric comorbidities [3,25,26].

We investigated potential predictors related to change in BMI and duration of inpatient stay. First*,* we examined *sociodemographic predictors* and found only an association between a greater change in BMI and living with both parents, which is quite similar to the results of previous studies [54,64]. Other studies postulated that patients living in a single parent household had higher drop-out rates and worse treatment outcomes [62,72,75]. Therefore, family variables seem to have an impact on treatment outcome, why the inclusion and support of family members in the treatment of adolescents with AN is essential, especially if difficulties and problems concerning family dynamics are observed.

Second, we investigated *clinical predictors* on BMI change and duration of inpatient stay. We observed that a lower BMI at admission was accompanied by higher change in BMI and longer duration of inpatient stay. Additionally, a longer duration of inpatient stay was associated with a higher BMI change. This can be explained by the fact, that patients with a lower BMI at admission need to gain more weight and therefore also require a longer duration of inpatient stay. Previous studies postulated that the BMI at admission as an indicator of illness severity is a predictor of treatment response in terms of a better prognosis in patients with higher BMI at admission and discharge [63,66]. Furthermore, higher levels of eating disorder psychopathology at admission were also associated with longer duration of inpatient stay, which is consistent with the current literature [33,34,36]. The severity of eating disorder symptoms associated with a low BMI at admission and possible medical complications often lead to a prolonged inpatient stay as they interfere with a continuous weight gain.

Third, we examined whether psychopharmacological treatment had an impact on the BMI change and the duration of inpatient treatment. We found that *antipsychotic medication* significantly predicted the BMI change, which could be due to the appetite-increasing side-effect of the psychotropic drug or to the influence of medication on the psychological symptoms of the disorder. This is an important result, as up to now there is still a controversial debate about the favorable influence of antipsychotic medication on weight gain or eating disorder-related psychological core symptoms [76,77,78,79,80,82,83,86]. However, in several studies the use of olanzapine (evidence grade B) [77,79,80,82,83] in adolescents or adults with AN was associated with greater weight gain and higher BMI post treatment (modest effects), while only in few studies beneficial effects for psychological symptoms such as obsessionality [80], intrusive anorectic thoughts [83], anxiety, and specific eating disorder symptoms [82] were observed. Furthermore, we found that *anxiolytic medication* predicted the length of hospital stay, which should be taken as a marker of higher illness severity of the patients receiving them. In particular, extreme fear of weight gain, anxiety before, during and after meals, and related difficulties of oral nutritional intake or suicidality led to administering anxiolytics for a short time at the beginning of treatment in this patient group, as there is no evidence to date that benzodiazepines contribute to weight development or eating disorder symptom improvement [78].

The predictive value of psychotropic medication on change in BMI and duration of inpatient treatment that we found, are important findings that expand the current literature, but must be interpreted with caution. To date, there is a limited evidence that AN-symptoms are favorable influenced by psychopharmaceuticals [25,26]. Thus, further studies are needed to clarify the impact of psychopharmacological medication on the treatment outcome of adolescents suffering from AN.

### Strengths and Limitations

The strengths of this study lie in its conduction in a conventional treatment environment with a relatively large “real-world” sample involving a wide spectrum from milder to extreme AN-cases. However, it should be noted that this study was conducted at a university hospital, where we often treat patients for whom other treatment strategies and facilities have already failed, who have high illness severity or sometimes even need to be admitted involuntarily, which may limit the comparability of our results with previous investigations that have include less severe AN-cases or patients highly motivated for treatment. Another strength of this study is the combined use of a gold-standard clinical interview and self-report instruments to evaluate the treatment outcomes.

Several limitations must be noted. First, this was an observational study longitudinally following an AN-cohort not including a control group. Thus, the study did not allow a comparison between the effectiveness of our treatment with other treatment approaches. Second, so far, we did not sufficiently collect long-term follow-up data, which means that the effectiveness of the present treatment approach cannot be evaluated with regard to the probability of relapse and long-term functioning. Third, a significant proportion of patients did not complete self-report instruments and the clinical interview at discharge (19.5–29.8% depending on the instrument and based on the number of patients who provided data at baseline), which was due to early discharge or study drop-out during inpatient treatment. This may also reflect the general difficulty in engaging this patient group into treatment and scientific studies. Forth, due to the low number of adolescents with binge-purging subtype of AN, we were not able to evaluate whether the effectiveness of our treatment differs with regard to the AN-subtype. Fifth, the generalizability of the results is limited by the inclusion of female patients only and the high percentage of included patients from families with high educational background.

## 5. Conclusions

To conclude, our findings are almost comparable to other studies having proven the effectiveness of a multimodal, disorder-specific, and age-adapted inpatient treatment approach for adolescents affected by AN with respect to change in BMI and eating disorder pathology e.g., [14,34,41,44]. However, as there is a lack in consensus how to define “favorable” or “unfavorable” treatment outcomes in previous studies, we recommend the general use of the newly (2013) defined DSM-5 remission criteria to improve comparability of outcomes across studies in future. The present study provides useful insights into some factors relevant for success of inpatient treatment in adolescents, for example that the use of antipsychotic medication may facilitate BMI gain, which may encourage further studies. More research is needed to identify moderating and mediating factors of the treatment course and factors that can predict relapse and long-term treatment success also including relevant psychological constructs such as temperament and personality, body image, family dynamics, and details on the use of psychotropic medication. This will help clarifying the question of which patients may face a more severe and enduring course of the illness despite getting similar treatments, and who can be helped best using which approach.

## Figures and Tables

**Table 1:** Sample description of female anorexia nervosa patients at admission (N = 126).

	Mean	SD	Range [Min, Max]
Age at admission	14.83	1.56	11–17
Age of eating disorder onset	13.83	1.71	8–17
Mean duration of illness (in months)	13.48	10.42	1–48
BMI at admission	14.40	1.32	10.50–17.42
Weight (kg) at admission	38.54	4.64	24.20–50.00
Height (m) at admission	1.63	0.07	1.41–1.77
Lowest weight since age of 12(kg)	36.83	4.48	24.00–46.70
Highest weight since age of 12 (kg)	50.79	8.59	28.15–76.00
Weight suppression (kg) ^1^	13.75	6.42	1.40–34.00
Age of mother	45.23	5.05	32–55
Age of father	48.75	6.09	36–67
	***N***	**%**	
Anorexia Nervosa subtype			
Restrictive	111	88.1%	
Binge/Purging	15	11.9%	
Anorexia Nervosa severity according to DSM-5 ^2^			
Mild	23	18.3%	
Moderate	30	23.8%	
Severe	33	26.2%	
Extreme	40	31.7%	
Psychiatric comorbidities (*N*, %Yes)			
Any	81	64.3%	
Obsessive-Compulsive Disorder	18	14.3%	
Depressive Disorder	49	38.9%	
Anxiety Disorder	9	7.1%	
Personality Disorder	5	4.0%	
Organic Brain Syndrome	16	12.7%	
Other	27	21.4%	
Medication use (*N*, %Yes)			
Antidepressant medication	55	43.7%	
Antipsychotics medication	79	62.7%	
Anxiolytic medication	25	19.8%	
Nutritional Supplements	126	100%	
Referral to clinic			
In-House outpatient clinic	57	45.2%	
Private practice of CAP or psychotherapist	18	14.3%	
Private practice of pediatrician	8	6.3%	
General practitioner	7	5.6%	
Other CAP clinic	5	4.0%	
Psychosomatic clinic	11	8.7%	
Pediatric clinic	8	6.3%	
Other outpatient center	12	9.5%	
Nationality			
Austria	120	95.2%	
Other	6	4.8%	
School type			
Grammar school	91	72.2%	
Vocational school (with A-level)	16	12.7%	
Vocational training school	4	3.2%	
Secondary modern school	13	10.4%	
Other school	2	1.6%	
Family status (living with…)			
both biological parents	82	65.1%	
single parent	43	34.7%	
no biological parent	1	0.8%	
Highest education mother/father			
University degree	44/51	37.0%/43.6%	
A level degree	24/18	20.2%/15.4%	
Below A-level degree	51/48	42.9%/41.0%	
Missing	7/9		
Marital status mother/father			
Married/in partnership	92/95	74.2%/78.5%	
Single	2/1	1.6%/0.8%	
Divorced/widowed	30/25	24.2%/20.6%	
Missing	2/5		
Number of siblings			
0	15	14.3%	
1	48	45.7%	
2	27	25.7%	
≥3	15	14.3%	
Missing	21	data ^1^	

^1^ Difference between maximum and minimum weight since age of 12; ^2^ DSM-5 severity definition based on BMI (percentiles) cut-off; Abbreviations: BMI Body-mass index, CAP Child and Adolescent Psychiatry.

**Table 2:** Differences in BMI and eating disorder pathology between admission and discharge including effect sizes.

Outcome Variable	Admission		Discharge		Test Statistic		Effect Size	
	Mean	SD	Mean	SD	*t* (df)	*p*	Cohen’s *d*	95% CI
BMI	14.40	1.32	17.01	1.29	20.269 (125)	**<0.001**	2.01	[1.67; 2.34]
BMI Percentile	1.47	3.41	12.77	11.97	11.812 (125)	**<0.001**	1.07	[0.84; 1.29]
EDE Total score	3.13	1.55	2.04	1.30	8.557 (95)	**<0.001**	0.75	[0.55; 0.94]
EDE Restraint ^a^	3.14	1.87	1.42	1.21	9.642 (95)	**<0.001**	1.06	[0.79; 1.33]
EDE Eating concern ^a^	2.62	1.60	1.48	1.25	7.677 (93)	**<0.001**	0.78	[0.55; 1.01]
EDE Weight concern ^a^	3.18	1.84	2.31	1.68	4.884 (95)	**<0.001**	0.49	[0.28; 0.70]
EDE Shape concern ^a^	3.55	1.63	2.98	1.71	3.940 (95)	**<0.001**	0.35	[0.17; 0.52]
EDE-Q Total score	2.88	1.63	1.83	1.39	6.861 (66)	**<0.001**	0.69	[0.47; 0.91]
EDE-Q Restraint ^a^	2.93	2.04	1.25	1.30	7.526 (65)	**<0.001**	0.94	[0.65; 1.24]
EDE-Q Eating concern ^a^	2.26	1.53	1.30	1.22	6.711 (66)	**<0.001**	0.68	[0.46; 0.90]
EDE-Q Weight concern ^a^	2.88	1.66	2.07	1.67	4.618 (65)	**<0.001**	0.49	[0.27; 0.71]
EDE-Q Shape concern ^a^	3.48	1.85	2.71	1.82	3.996 (66)	**<0.001**	0.42	[0.20; 0.64]
EDI-2 Global score	68.73	36.90	54.22	37.18	3.962 (66)	**<0.001**	0.39	[0.19; 0.59]
EDI-2 Drive for thinness ^b^	8.55	7.20	6.07	7.13	3.210 (66)	**0.002**	0.35	[0.13; 0.57]
EDI-2 Bulimia ^b^	1.18	2.70	0.59	2.10	2.012 (65)	0.048	0.24	[0.00; 0.48]
EDI-2 Body dissatisfaction ^b^	11.48	7.86	10.45	8.65	1.085 (66)	0.282	0.12	[−0.10; 0.35]
EDI-2 Ineffectiveness ^b^	6.97	6.12	5.90	6.29	1.842 (66)	0.070	0.17	[−0.01; 0.36]
EDI-2 Perfectionism ^b^	6.18	4.27	6.04	4.53	0.330 (66)	0.742	0.03	[−0.15; 0.21]
EDI-2 Interpersonal distrust ^b^	4.72	4.49	3.39	3.81	3.856 (66)	**<0.001**	0.31	[0.15; 0.48]
EDI-2 Interoceptive awareness ^b^	6.78	5.85	4.32	4.91	4.008 (66)	**<0.001**	0.45	[0.22; 0.69]
EDI-2 Maturity fears ^b^	7.42	5.22	5.85	4.94	2.541 (66)	0.013	0.31	[0.06; 0.55]
EDI-2 Asceticism ^b^	5.57	4.53	4.57	4.83	2.041 (66)	0.045	0.21	[0.00; 0.42]
EDI-2 Impulse regulation ^b^	3.71	4.54	2.21	3.57	3.217 (66)	**0.002**	0.36	[0.13; 0.59]
EDI-2 Social insecurity ^b^	6.20	4.36	4.80	4.27	3.553 (66)	**0.001**	0.32	[0.14; 0.51]

^a^ tested on a Bonferroni-corrected significance level of α = 0.0125; ^b^ tested on a Bonferroni-corrected significance level of α = 0.0045; Abbreviations: BMI Body Mass Index, EDE Eating Disorder Examination Interview, EDE-Q Eating Disorder Examination Questionnaire, EDI-2 Eating Disorder Inventory-2.

**Table 3:** Differences between remission groups regarding demographic and clinical characteristics.

	No Remission (*N* = 45) a	Partial Remission (*N* = 32) b	Full Remission (*N* = 24) c	Test Statistic	Post-Hoc Analyses
	Mean (SD)	Mean (SD)	Mean (SD)	*F*(df), *p*	
Age at admission	15.62 (1.32)	14.23 (1.43)	14.57 (1.88)	8.900 (2,96), **<0.001**	a > b,c
BMI at admission	14.19 (1.11)	14.71 (1.53)	14.19 (1.44)	1.654 (2,96), 0.197	
BMI percentile at admission	0.44 (1.27)	2.24 (2.97)	1.68 (4.20)	4.249 (2,96), **0.017**	a < b
EDE total at admission	3.47 (1.58)	3.39 (1.31)	2.16 (1.44)	6.652 (2,93), **0.002**	c < a,b
EDE-Q total at admission	3.39 (1.45)	2.94 (1.48)	2.01 (1.40)	5.874 (2,78), **0.004**	c < a
EDI-2 total at admission	76.68 (36.75)	71.51 (36.19)	52.68 (30.76)	3.206 (2,81), **0.046**	c < a
Age of eating disorder onset	14.62 (1.27)	13.29 (1.74)	13.52 (2.02)	7.284 (2,96), **0.001**	a > b,c
Duration of illness	13.56 (9.75)	12.05 (10.71)	14.37 (10.29)	0.375 (2,96), 0.688	
Duration of inpatient stay	66.53 (35.48)	81.60 (29.39)	66.35 (30.53)	2.243 (2,95), 0.112	
Weight suppression	14.30 (6.22)	12.98 (4.91)	14.50 (9.04)	0.459 (2,95), 0.633	
	**%**	**%**	**%**	**Chi^2^ (df), *p***	
Any psychiatric comorbidity	62.2%	74.2%	52.2%	2.838(2), 0.242	
Antidepressant medication	42.2%	61.3%	26.1%	6.792(2), **0.034**	b > c
Antipsychotic medication	62.2%	71.0%	52.2%	1.998(2), 368	
Anxiolytic medication	17.8%	29.0%	17.4%	1.653(2), 0.438	
Single parent families	42.2%	29.0%	21.7%	3.249(2), 0.197	

Abbreviations: BMI Body Mass Index, EDE Eating Disorder Examination Interview, EDE-Q Eating Disorder Examination Questionnaire, EDI-2 Eating Disorder Inventory-2. Group specifications: a = refers to no remission group; b = refers to partial remission group and c = refers to full remission group.

**Table 4:** Results of multivariate linear regression analyses predicting change in BMI and duration of inpatient stay.

Predictor	b(SE)	Beta	*t*	*p*
Outcome: Change in BMI (R^2^ = 0.58; adjusted R^2^ = 0.56) ^3^				
Constant	7.449 (1.11)		6.704	**<0.001**
**Sociodemographic predictors**				
Family status ^2^	0.452 (0.18)	0.148	2.460	**0.015**
**Clinical predictors**				
BMI at admission	−0.504 (0.07)	−0.460	−7.506	**<0.001**
Duration of inpatient stay	0.020 (<0.01)	−0.459	7.005	**<0.001**
**Psychopharmacological predictors**				
Antipsychotic medication ^1^	0.439 (0.19)	0.146	2.288	**0.024**
Anxiolytic medication ^1^	−0.111 (0.24)	−0.030	−0.459	0.647
**Outcome: Duration of inpatient stay (R^2^ = 0.26; adjusted R^2^ = 0.22) ^3,4^**				
Constant	149.358 (31.06)		4.809	**<0.001**
**Clinical predictors**				
BMI at admission	−7.250 (2.15)	−0.307	−3.374	**0.001**
EDE total at admission	5.440 (1.90)	0.259	2.858	**0.005**
**Psychopharmacological predictors**				
Antidepressant medication ^1^	10.660 (5.91)	0.169	1.803	0.074
Antipsychotic medication ^1^	5.219 (6.11)	0.080	0.854	0.395
Anxiolytic medication ^1^	18.204 (7.14)	0.233	2.549	**0.012**

^1^ coded as follows: 0 = no, 1 = yes; ^2^ coded as follows: 1 = single parent families, 2 = living with both biological parents; ^3^ predictors with *p* < 0.10 in univariate analyses considered as predictors for multivariate analyses; ^4^ only cases with regular discharge and duration of inpatient stay < 300 considered for this analysis (N = 105); Abbreviations: BMI Body-Mass-Index; EDE-I Eating Disorder Examination Interview; Note: Only predictors with *p* < 0.10 in the univariate analyses were considered for these multivariate regression analyses.

## Data Availability

The data that support the findings of this study are available on request from the corresponding author.

## Supplementary Materials

The following are available online at https://www.mdpi.com/article/10.3390/jcm10143190/s1. Table S1: Description of the treatment phases of the Viennese Treatment-Concept for Adolescents Suffering from Anorexia Nervosa (ViTAA), Table S2: Bivariate correlations between eating disorder outcome instruments (total scores) at admission and discharge, Table S3 Results of univariate linear regression analyses predicting change in BMI, Table S4: Results of univariate linear regression analyses predicting duration of inpatient stay, Figure S1: Bivariate correlations between EDE, EDE-Q and EDI-2 total and subscores at admission.
