# Supplementary material for: Short-Term Outcome of Inpatient Treatment for Adolescents with Anorexia Nervosa Using DSM-5 Remission Criteria
Source: J Clin Med. 2021 Jul 20;10(14):3190. doi: 10.3390/jcm10143190 (PMC8307185; doi:10.3390/jcm10143190)
Supplement: Supplementary file 1 [file jcm-10-03190-s001.zip › jcm-1293585-supplementary.pdf]

Table S1. Description of the treatment phases of the Viennese Treatment-Concept for Adolescents Suffering from Anorexia Nervosa (ViTAA)

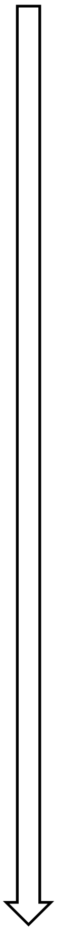

Phase	Description / Goals
1. Orientation phase (first week)	This phase includes somatic clarification, diagnostic and treatment planning by a multi-professional team including<br>- full spectrum of clinical and apparatus based diagnostic procedures like cranial magnetic resonance imaging (cMRI), electroencephalo-graphy (EEG), detailed laboratory diagnostic, electrocardiogram (ECG), cardiac ultrasound, bone-densitometry, height and weight assessment and further somatic clarification in collaboration with pediatrics if necessary,<br>- detailed psychological diagnostic, physiotherapeutic and occupational therapeutic diagnostic<br>- further diagnostic examinations if required (e.g.. logopaedic diagnostic, family interaction diagnostic).<br>In this phase food and fluid in-take of the patient in own responsibility is observed.
2. Learning phase A (‘Building-up’)	This phase is characterized by a focus on weight gain as well as regaining autonomy over food preparation and consumption. Nutritional rehabilitation takes place under provision of supervised meals through specially trained nurses and is individually adapted in consultation with clinical nutritionist. Furthermore, medication with food supplements is administered and psychotropic drugs are considered depending on the eating disorder severity and treatment course as well as existing comorbidities.
3. Learning phase B (‘Implementing’)	This phase is characterized by gradual promotion of personal responsibility regarding food and fluid intake with increasing focus on main psychological symptoms.
4. Learning phase C (‘Stabilizing’)	In this phase, the patients should reach and hold their discharge weight with emphasis on outdoor orientation and reintegration. Regular discharge takes place when the discharge weight is reached, which is determined in the course of treatment on the basis of individual age- and sex-dependent BMI-percentile curves, taking into account the individual growth and weight trajectory since birth.
5. Intensive Phase<br>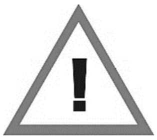	This phase describes the procedure in case of vital-threat because of malnutrition and associated somatic complications like severe bradycardia, pericardial effusion, electrolyte abnormalities, hypotension, edema, dehydration, hypothermia, hypoproteinemia risk of developing a refeeding syndrome. Depending on the severity of somatic complications constant medical observation including monitoring of the vital parameters, infusion therapy, naso-gastric tube feeding, continuous labor controls and additional examinations are carried out. If serious life-threatening somatic complications obscure patients are transferred to a pediatric ward until the stabilization of the somatic condition is reached.

Table S2. Bivariate correlations between eating disorder outcome instruments (total scores) at admission and discharge

Admission			
	**EDE total**	**EDE-Q total**	**EDI-2 total**
**EDE total**	-		
**EDE-Q total**	.791***	-	
**EDI-2 total**	.716***	.710***	-
Discharge			
	**EDE total**	**EDE-Q total**	**EDI-2 total**
**EDE total**	-		
**EDE-Q total**	.765***	-	
**EDI-2 total**	.621***	.708***	-

Abbreviations: EDE Eating Disorder Examination Interview, EDE-Q Eating Disorder Examination Questionnaire, EDI-2 Eating Disorder Inventory-2; *** p <.001

Figure S1. Bivariate correlations between EDE, EDE-Q and EDI-2 total and subscores at admission

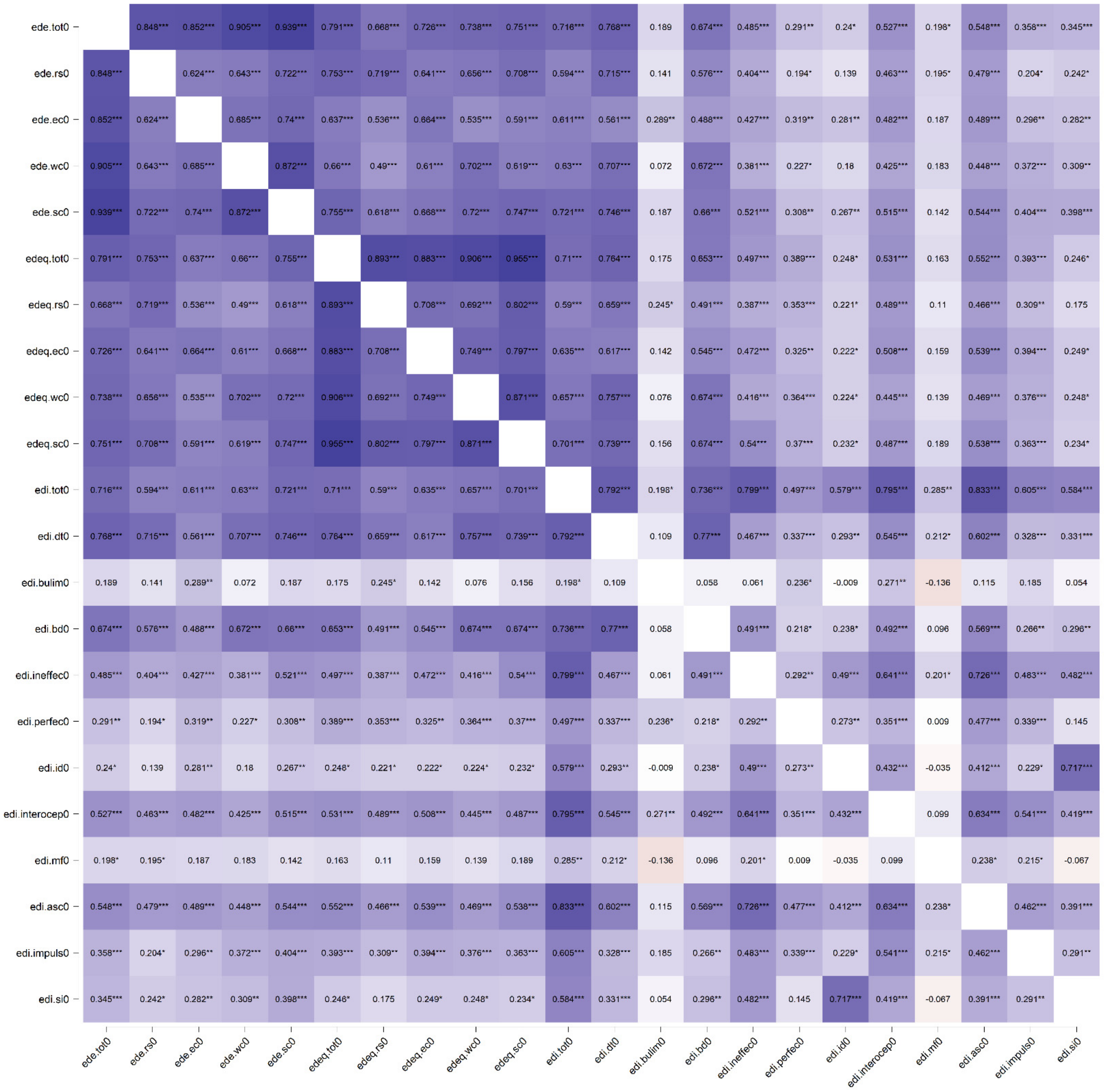

Abbreviations:

ede.tot0 EDE Total score
ede.rs0 EDE Restraint score
ede.ec0 EDE Eating concern score
ede.wc0 EDE Weight concern score
ede.sc0 EDE Shape concern score
edeq.tot0 EDEQ Total score
edeq.rs0 EDEQ Restraint score
edeq.ec0 EDEQ Eating concern score
edeq.wc0 EDEQ Weight concern score
edeq.sc0 EDEQ Shape concerns score
edi.tot0 EDI-2 Total score
edi.dt0 EDI-2 Drive for thinness score
edi.bulim0 EDI-2 Bulimia score
edi.bd0 EDI-2 Body dissatisfaction score
edi.ineffec0 EDI-2 Ineffectiveness score
edi.perfec0 EDI-2 Perfectionism score
edi.id0 EDI-2 Interpersonal Distrust Score
edi.interocep0 EDI-2 Interoceptive awareness score
edi.mf0 EDI-2 Maturity fears score
edi.asc0 EDI-2 Asceticism score
edi.impuls0 EDI-2 Impulse regulation score
edi.si0 EDI-2 Social insecurity score

EDE Eating Disorder Examination Interview
EDE-Q Eating Disorder Examination Questionnaire
EDI-2 Eating Disorder Inventory-2

Table S3. Results of univariate linear regression analyses predicting change in BMI (slopes presented only)

**Predictor**	***b*(SE)**	**Beta**	***t*-value**	***p***
Sociodemographic:				
Age at admission	-0.013 (0.08)	-0.014	-0.159	.874
Family status[1]	0.479 (0.27)	0.158	1.786	.077
Clinical:				
BMI at admission	-0.625 (0.08)	-0.568	-7.690	**<.001**
EDE total at admission	0.066 (0.09)	0.068	0.749	.456
Age of eating disorder onset	0.028 (0.08)	0.033	0.369	.713
Duration of illness	-0.012 (0.01)	-0.083	-0.926	.356
Weight suppression	0.028 (0.02)	0.125	1.381	.170
Duration of inpatient stay	0.024 (< 0.01)	0.568	7.620	**<.001**
Any psychiatric comorbidity[2]	-0.043 (0.27)	-0.014	-0.161	.873
Psychopharmacological:				
Antidepressant medication[2]	-0.200 (0.26)	-0.069	-0.766	.445
Antipsychotic medication[2]	0.773 (0.26)	0.259	2.988	**.003**
Anxiolytic medication[2]	0.579 (0.32)	0.160	1.805	.074

[1] coded as follows: 1 = single parent families, 2 = living with both biological parents; [2] coded as follows: 0 = no, 1 = yes; Abbreviations: BMI Body-Mass-Index; EDE Eating Disorder Examination Interview

Table S4. Results of univariate linear regression analyses predicting duration of inpatient stay

Predictor	*b*(SE)	Beta	*t*-value	*p*
Sociodemographic:				
Age at admission	1.486 (1.97)	0.073	0.754	.453
Family status[1]	-0.740 (6.43)	-0.011	-0.115	.908
Clinical:				
BMI at admission	-5.079 (2.26)	-0.213	-2.246	**.027**
EDE total at admission	6.276 (1.97)	0.299	3.180	**.002**
Age of eating disorder onset	0.280 (1.78)	0.015	0.157	.875
Weight suppression	0.586 (0.48)	0.119	1.217	.226
Duration of illness	0.396 (0.30)	0.129	1.339	.183
Any psychiatric comorbidity[2]	8.016 (6.38)	0.121	1.257	.212
Psychopharmacological:				
Antidepressant medication[2]	12.648 (6.03)	0.200	2.099	**.038**
Antipsychotic medication[2]	16.619 (6.18)	0.253	2.690	**.008**
Anxiolytic medication[2]	23.511 (7.22)	0.302	3.257	**.002**

[1] coded as follows: 1 = single parent families, 2 = living with both biological parents; [2] coded as follows: 0 = no, 1 = yes; Abbreviations: BMI Body-Mass-Index; EDE Eating Disorder Examination Interview; Note: only cases with regular discharge and duration of inpatient stay < 300 days considered for this analysis (N = 108)
